# Overexpression of *StDREB2* Transcription Factor Enhances Drought Stress Tolerance in Cotton (*Gossypium barbadense* L.)

**DOI:** 10.3390/genes10020142

**Published:** 2019-02-14

**Authors:** Mohamed A. El-Esawi, Aisha A. Alayafi

**Affiliations:** 1 Botany Department, Faculty of Science, Tanta University, Tanta 31527, Egypt; 2 Biological Sciences Department, Faculty of Science, University of Jeddah, Jeddah 21577, Saudi Arabia; aal_shareaf@hotmail.com

**Keywords:** *StDREB2*, transgenic cotton, drought, antioxidants, osmolytes, genes expression

## Abstract

Drought stress significantly restricts plant growth and crop productivity. Cotton is the most important textile fiber and oilseed crop worldwide, and its cultivation is affected by drought stress, particularly in dry regions. Improving cotton tolerance to drought stress using the advanced genetic engineering technologies is a promising strategy to maintain crop production and fiber quality and meet the increasing worldwide fiber and oil demand. Dehydration-responsive element binding (DREB) transcription factors play a main role in regulating stresses-tolerance pathways in plant. This study investigated whether potato *DREB2* (*StDREB2*) overexpression can improve drought tolerance in cotton. *StDREB2* transcription factor was isolated and overexpressed in cotton. Plant biomass, boll number, relative water content, soluble sugars content, soluble protein content, chlorophyll content, proline content, gas-exchange parameters, and antioxidants enzymes (POD, CAT, SOD, GST) activity of the *StDREB2*-overexpressing cotton plants were higher than those of wild type plants. By contrast, the contents of malondialdehyde, hydrogen peroxide and superoxide anion of StDREB2-overexpressing transgenic plants were significantly lower than that of the wild type plants. Moreover, the transgenic cotton lines revealed higher expression levels of antioxidant genes (*SOD*, *CAT*, *POD, GST*) and stress-tolerant genes (*GhERF2*, *GhNAC3*, *GhRD22*, *GhDREB1A*, *GhDREB1B*, *GhDREB1C*) compared to wild-type plants. Taken together, these findings showed that *StDREB2* overexpression augments drought stress tolerance in cotton by inducing plant biomass, gas-exchange characteristics, reactive oxygen species (ROS) scavenging, antioxidant enzymes activities, osmolytes accumulation, and expression of stress-related genes. As a result, *StDREB2* could be an important candidate gene for drought-tolerant cotton breeding.

## 1. Introduction

Abiotic stresses, such as salinity, drought, and high or low temperature significantly mitigate plant performance and development worldwide [1]. Particularly, drought is one of the major factors that damages numerous physiological processes, resulting in significant reductions in crop growth and productivity [2]. In addition, drought stress stimulates reactive oxygen species (ROS) accumulation, as well as oxidative damage in plant species. ROS accumulation also influences antioxidant systems, osmolytes, and other macromolecules in plants [3,4,5,6]. To alleviate the drought stress-induced negative impacts and scavenge ROS accumulation, plants manipulate different physiological processes such as osmolytes biosynthesis, photosynthesis, water uptake, hormonal metabolism, and induction of enzymatic (peroxidase, ascorbate peroxidase, superoxide dismutase and catalase) and non-enzymatic (ascorbic acid and glutathione) antioxidant systems [7,8].

Various strategies have been applied to improve plant growth and alleviate the negative impacts of abiotic stresses. These approaches included the use of plant growth regulators and biostimulants such as phytohormones, plant growth promoting rhizobacteria and seaweeds [9,10,11,12,13,14,15,16,17,18,19,20,21,22,23]. Furthermore, the approach of genetic engineering and regulation of stress-related genes expression has shown its great potential in enhancing crop yield and tolerance to severe environmental stresses including drought [8,24,25,26,27,28]. Drought stress-induced genes comprise two groups divided based on their product functions in plants [8]. The functional proteins belong to the first group and include the detoxification enzymes, membrane proteins, osmolytes biosynthesis enzymes, and proteins mediating macromolecules protection, while the second group includes the regulatory proteins such as proteinases, protein kinases, and transcription factors that regulate genes expression and signal transductions [8,29]. Many transcription factors have been identified and showed potential in enhancing plant tolerance to different harmful stresses through regulating the expression of the downstream stress-related genes [30,31,32,33,34,35,36]. Dehydration-responsive element binding (DREB) transcription factors belong to the AP2/ERF family and comprise *DREB1* and *DREB2*, which play a main role in regulating stress-tolerance pathways in plants [30,31]. DREB1 was first cloned from *Arabidopsis* and revealed high levels of drought stress tolerance when overexpressed in crops [32]. Overexpression of *StDREB1* in potato enhanced tolerance to drought, salt, oxidative, and cadmium stresses [33,34,35]. Moreover, *StDREB2* overexpression in potato improved tolerance to salt and cadmium stress [35,36].

Cotton (*Gossypium barbadense* L.) is the most essential textile fibers and oilseed crop worldwide [37,38]. Cultivation of cotton consumes high quantities of water resources that are currently limited, particularly in dry regions such as Egypt, China, and Uzbekistan [38]. Moreover, cotton crop has a variable degree of sensitivity to drought [39,40]. Therefore, it is essential to augment drought tolerance in cotton to meet the increasing global demands. Previous reports demonstrated the importance of using the transgenic approaches in improving cotton drought tolerance. For example, Yu et al. [38] reported that *Arabidopsis EDT1/HDG11* overexpression in cotton enhanced drought tolerance. Liu et al. [28] also stated that rice SNAC1 overexpression augmented drought tolerance in cotton. Moreover, higher drought tolerance level has been recorded in cotton plants overexpressing *AmDUF1517* [41], *ScALDH21* [42], *GhAnn1* [43], or *OsSIZ1* [44]. However, developing new drought-tolerant cotton genotypes is still needed to overcome the limited water resources and adverse drought impacts, maintain productivity and fiber quality and meet the increasing fiber and oil needs worldwide. The current study, therefore, investigated whether the overexpression of *StDREB2* transcription factor, isolated from *Solanum tuberosum* L., can improve drought tolerance in cotton. Molecular analysis of the wild type and overexpressed cotton lines were performed. Various physiological attributes and the expression analysis of stresses-related genes were studied in the wild type and transgenic plants to evaluate cotton transgenics performance under drought stress conditions. Results of the current study would help to demonstrate a novel role for *StDREB2* in mediating drought stress tolerance in plants.

## 2. Materials and Methods

### 2.1. Plant Material and Growth Conditions

Cotton (*Gossypium barbadense* L. cv. Giza 86) seeds and potato (*Solanum tuberosum* L. cv. Spunta) seed tubers obtained from Agricultural Research Centre in Egypt were utilized in current investigation. Cotton seeds were surface-sterilized using sodium hypochlorite (10%) for 6 min, immersed in sterile H_2_O five times, and then left to grow on a wet towel for 7 days, whereas potato tubers were left at 20 °C in the dark till sprouting. The germinated cotton seedlings and sprouted potato tubers were then planted in pots containing soil mixtures composed of perlite, sand and peat (1:1:1, *v*/*v*/*v*) and kept to grow with regular watering under adjusted conditions of 27/19 °C, 14/10 h and humidity of 75%.

### 2.2. Vector Construction and Cotton Transformation

RNeasy Plant Mini kit (Qiagen, Hilden, Germany) was used to extract the total RNA from potato young plantlets. RNase-Free DNase Set (Qiagen) was then used to remove the contaminating DNA. Reverse Transcription kit (Qiagen) was used to synthesize cDNA. The full length cDNA of *StDREB2* was amplified and cloned using Gateway cloning technology (Invitrogen, Waltham, MA, USA) as previously described in detail by Bouaziz at al. [36]. Briefly, *StDREB2* cDNA was inserted into a pMDC32 vector (Invitrogen). The resulting constructs (pMDC32::*StDREB2*) were then transferred into *Agrobacterium tumefaciens* (EHA105 strain) which served to transform cotton Giza 86 plants following the *Agrobacterium*-mediated transformation methodology [45].

### 2.3. Validation Analysis of Transgenic Cotton Lines

T_0_ and T3 homozygous overexpressed cotton plants were validated by evaluating the expression levels of *StDREB2* in the positive transgenic cotton lines using quantitative real-time PCR (qRT-PCR) analysis. RNA extractions and cDNA syntheses were carried out from the wild type and T_0_ and T_3_ overexpressed cotton lines as stated above. qRT-PCR reactions were achieved in triplicates (three biological) using QuantiTect SYBR Green PCR kit (Qiagen) according to the manufacturer’s protocol. qRT-PCR condition was adjusted as described by Bouaziz at al. [36]. Additionally, the specific gene primers previously designed for *StDREB2* and the internal reference *ef1α* (elongation factor) [36] were used. *StDREB2* expression level was estimated following the 2^−ΔΔCt^ method [46].

### 2.4. Plant Growth and Drought Stress Treatment

The seedlings of wild-type and three different T_3_ homozygous *StDREB2*-overexpressing transgenic cotton lines (OX-3, OX-7, OX-12) were transplanted into pots comprising the aforementioned soil mixture and kept to grow with daily watering under the same condition for four weeks. The cotton plants were then divided into two groups as follows; (i) control, 27/19 °C with regular daily watering; and (ii) drought stress, 27/19 °C without watering. All treatments lasted for two weeks, and plant samples were then collected for further analyses. The experiments were performed in five replicates.

### 2.5. Estimation of Boll Number, Plant Biomass, and Relative Water Content

The collected plants were washed and boll number per plant was calculated. Shoot and root samples were oven dried at 80 °C for 72 h to estimate their dry weights. Leaf relative water content (RWC) was estimated as previously stated by Yamasaki and Dillenburg [47].

### 2.6. Estimation of Chlorophyll, Proline, Soluble Sugar, and Soluble Protein Contents

To estimate the leaf chlorophyll content, 0.1 g fresh leafy tissue was completely mixed with dimethyl sulfoxide and left in the dark for a couple of days. The homogenate absorbance was spectrophotometrically taken at 645 and 663 nm as previously described by Arnon [48]. Leaf-free proline content was determined using the protocol of Bates et al. [49], and absorbance was taken at 520 nm using toluene as a blank. To estimate the contents of leaf soluble proteins and sugars, leafy samples were homogenized in a 100 mM Tris buffer (1 mL) and then centrifuged at high speed for 12 min. Soluble sugar content was then estimated according to the protocol of Dey [50]. Soluble protein content was also measured as previously stated by Bradford [51].

### 2.7. Determination of Gas-Exchange Parameters

The net photosynthesis rate (*P_n_*), transpiration rate (*E*) and stomatal conductance (*g_s_*) were determined in leaf using a gas exchange system (ADC BioScientific, U.K.) at 9:30 a.m. as previously described by Holá et al. [52].

### 2.8. Estimation of Contents of Hydrogen Peroxide, Superoxide Anion, and Malondialdehyde

The content of leaf hydrogen peroxide (H_2_O_2_) was assayed by extracting 50 mg of leafy tissue in 0.5 mL of 0.1% TCA, followed by centrifugation at the highest speed. H_2_O_2_ content was quantified as reported by Velikova et al. [53]. The content of superoxide anion (O_2_^−^) in leaf was quantified as previously described by Esim et al. [54]. The content of leaf lipid peroxidation in terms of malondialdehyde (MDA) was determined as previously stated by Rao and Sresty [55].

### 2.9. Estimation of Antioxidant Enzyme Activities

Leaf tissue (0.5 g) was homogenized in 0.1 M phosphate buffer (pH 7.5) and 0.5 mM EDTA. The extract was filtered and centrifuged for 12 min at 13,000 × *g* and 4 °C. The supernatant was applied for antioxidant enzymes analyses. Catalase (CAT) enzyme activity was assayed as previously stated by Aebi [56], and absorbance was then read at 240 nm. Superoxide dismutase (SOD) and peroxidase (POD) activities were determined as previously described by Zhang [57]. The activity of glutathione-*S*-transferase (GST) was determined as reported by Hasanuzzaman and Fujita [58], and absorbance was measured at 340 nm. The activity of antioxidant enzymes was expressed as unit per milligram protein (EU mg^−1^ protein).

### 2.10. Expression Analysis of Antioxidant and Stress-Related Genes

Quantitative real-time PCR analysis was used to determine the expression of antioxidant enzymes genes (*CAT, SOD, POD*, *GST*) and stress-tolerant genes (*GhERF2, GhNAC3, GhRD22, GhDREB1A, GhDREB1B, GhDREB1C*) in wild type and T_3_ *StDREB2*-overexpressing cotton lines grown under normal and drought conditions. Isolation of RNA and synthesis of cDNA from plant tissues were carried out as mentioned above. qRT-PCR reactions were performed in triplicates (three biological and three technical replicates) using a QuantiTect SYBR Green PCR kit according to the manufacturer protocols. qRT-PCR condition was adjusted as described by Hao et al. [41]. The primers previously designed for the genes assayed [41] were used in amplification. *Small-subunit rRNA* was used as an internal control [41]. Relative gene expression level was estimated using 2^−ΔΔCt^ method.

### 2.11. Statistical Analysis

Data were analyzed and a one-way analysis of variance (ANOVA) was carried out using SPSS version 16.0. Data represent the means ± standard error (SE) (*n* = 5) and are significantly different at *p* ≤  0.05.

## 3. Results and Discussion

### 3.1. Transformation and Analysis of Transgenic Cotton Lines

**Figure 1:**
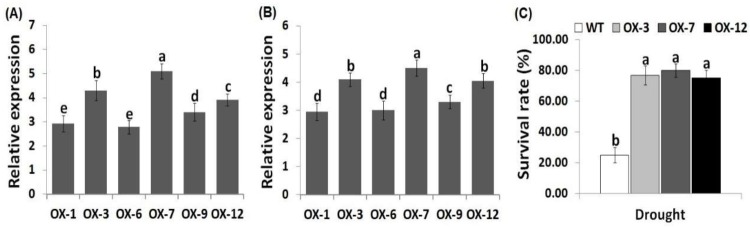
Molecular analyses and survival rate of cotton lines. *StDREB2* expression in T_0_ (**A**) and T_3_ (**B**) transgenic cotton lines using qRT-PCR. Survival rate of the wild type and transgenic lines following 12-day recovery after drought treatment (**C**).

Cotton represents the most essential textile fiber and oilseed crop worldwide [37,38]. Its cultivation is adversely influenced by drought stress, particularly in dry regions. Therefore, developing new drought-tolerant cotton genotypes using the currently advanced genetic engineering technologies are needed. As a result, to augment the drought tolerance of cotton in the present study, potato *StDREB2* gene was overexpressed in cotton, resulting in the generation of eighteen *StDREB2*-overexpressing transgenic lines. Exogenous *StDREB2* transcription level was validated in six T_0_ and T_3_ transgenic cotton lines (OX-1, OX-3, OX-6, OX-7, OX-9, and OX-12) using quantitative RT-PCR (Figure 1A,B). Three T_3_ transgenic cotton lines (OX-3, OX-7, OX-12) showed the highest *StDREB2* expression level and were utilized for further analyses. Following the drought stress treatment, a 12-day growth recovery was performed. Survivals rate of the transgenic cotton genotypes (OX-3, OX-7, OX-12) was significantly higher than that of the wild type plants (Figure 1C).

### 3.2. StDREB2 Overexpression Enhances Cotton Biomass, Boll Number, and Relative Water Content under Drought Stress Conditions

**Figure 2:**
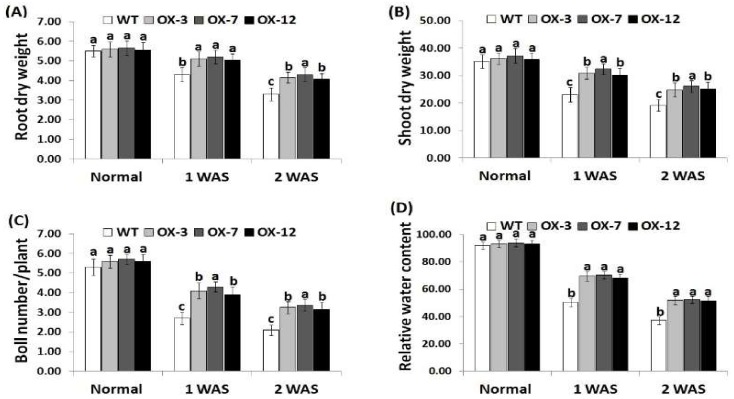
Root dry weight (g/plant) (**A**), shoot dry weight (g/plant) (**B**), boll number per plant (**C**), and relative water content (%) (**D**) of wild-type and *StDREB2*-overexpressing cotton lines under normal and drought conditions. WAS represents week after drought stress. Data represent means ± SE (*n* = 5). Same letters on columns tops denote non-significant differences (*p* ≤ 0.05).

Leaf-relative water content represents a main indicator for water status balance in plant cells [59,60]. Reduction in the relative water content induces osmotic stress and ultimately affect crop growth, biomass, and development [61]. The effect of *StDREB2* overexpression on the growth, biomass, and leaf-relative water content of cotton plants has been investigated in the current study. There was no significant difference in root dry weight, shoot dry weight, boll number per plant, or RWC between the wild type and *StDREB2*-overexpressing cotton plants under normal condition (Figure 2A–D). However, under drought condition, reduction in cotton growth, biomass, and RWC was noticed for the wild type and overexpressed cotton compared with normal condition. The greatest reductions were recorded at 2 WAS (week after drought stress). Nevertheless, under drought conditions, cotton transgenic plants revealed significantly higher growth, biomass, and RWC compared with wild type plants (Figure 2A–D). The results demonstrated that *StDREB2* overexpression in cotton augments their growth, biomass, RWC, and drought tolerance. These advantages might confer a greater survival rate in harsh environments with limited water resources. These findings are in agreement with previous studies that revealed higher plant biomass, boll number, and RWC in cotton plants overexpressing stress-tolerant genes compared with wild type plants [28,41].

### 3.3. StDREB2 Overexpression in Cotton Increases Chlorophyll and Osmolytes Contents under Stress

**Figure 3:**
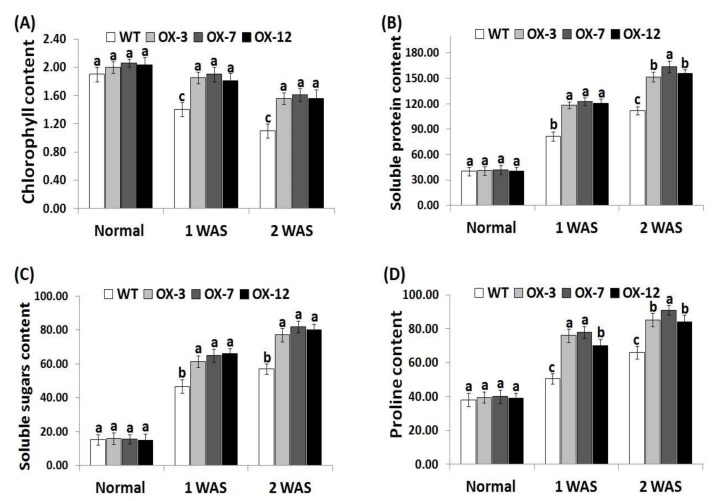
Chlorophyll content (mg g^−1^ FW) (**A**), soluble protein content (mg g^−1^ FW) (**B**), soluble sugar content (mg g^−1^ FW) (**C**), and proline content (µg g^−1^ FW) (**D**) of wild type and *StDREB2*-overexpressing cotton under normal and drought conditions. FW represents fresh weight. Data are means ± SE (*n* = 5). Same letters on columns denote non-significant differences (*p* ≤ 0.05).

Due to its essential role in photosynthesis, chlorophyll content is an useful indicator for investigating drought tolerance in plants [62]. In addition, soluble sugars, soluble proteins, free proline, and other osmolytes play a key role as ROS scavengers and osmoprotectants to maintain osmotic adjustment in plant cells under stress conditions [63,64]. Soluble sugars and proteins significantly reduce dehydration in plant cells and help maintain the cellular macromolecules’ function [65]. Therefore, the changes in levels of chlorophyll, soluble sugar, soluble proteins, and proline were recorded in the wild type and *StDREB2*-overexpressing cotton plants under different conditions (Figure 3A–D). No significant difference was observed in the levels of chlorophyll, soluble sugars, soluble proteins, and proline between wild type and overexpressed cotton lines under normal condition (Figure 3A–D). However, remarkable reductions in chlorophyll content and increases in osmolytes level were observed for the wild-type and *StDREB2*-overexpressing genotypes under drought conditions as compared to normal conditions. Nevertheless, *StDREB2*-overexpressing genotypes accumulated significantly higher levels of chlorophyll and osmolytes compared with the wild type plants under drought condition (Figure 3D), indicating that the transgenic genotypes were better capable to maintain their chlorophyll and osmolytes contents than the wild type. These results also suggest that *StDREB2* overexpression conferred the transgenic cotton lines higher osmoregulation ability to combat the drought-induced dehydration stress. These findings are also in agreement with previous studies that revealed higher chlorophyll content and osmolyte levels in cotton crop overexpressing stress-tolerant genes compared to wild-type [38,41].

### 3.4. StDREB2 Overexpression in Cotton Promotes Gas-Exchange Parameters under Stress Conditions

**Figure 4:**
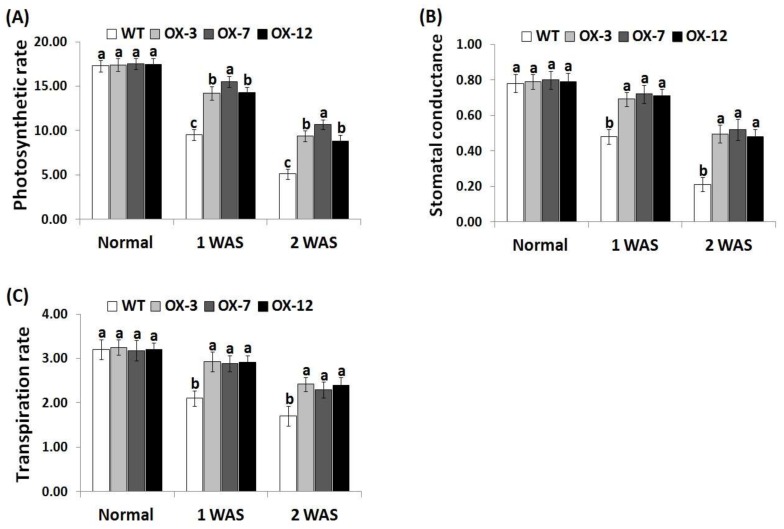
Photosynthetic rate (*P_n_*, μmol m^2^ s^−1^) (**A**)*,* stomatal conductance (*g_s_*, mol m^2^ s^−1^) (**B**), and transpiration rate (*E*, mmol m^2^ s^−1^) (**C**) of wild type and *StDREB2*-overexpressing cotton lines under normal and drought conditions. WAS represents week after drought stress. Data represent means ± SE (*n* = 5). Same letters on columns tops denote non-significant differences (*p* ≤ 0.05).

To investigate whether *StDREB2* overexpression in cotton can modulate gas-exchange attributes under drought stress, stomatal conductance, and photosynthetic and transpiration rates were determined in the wild type and *StDREB2*-overexpressing cotton under drought condition (Figure 4A–C). *StDREB2*-overexpressing genotypes did not display obvious differences in gas-exchange parameters under normal conditions as compared with the wild type plants (Figure 4A–C). By contrast, remarkable increases in gas-exchange attributes were reported for wild type and overexpressed plants under drought conditions compared with normal condition. Under drought conditions, gas-exchange parameters of transgenic cotton genotypes were remarkably higher than that of wild type, suggesting that the photosynthetic system of the overexpressed lines was less inhibited by drought stress as compared to the wild type.

### 3.5. StDREB2 Overexpression in Cotton Decreases Oxidative Stress Markers under Drought Stress

**Figure 5:**
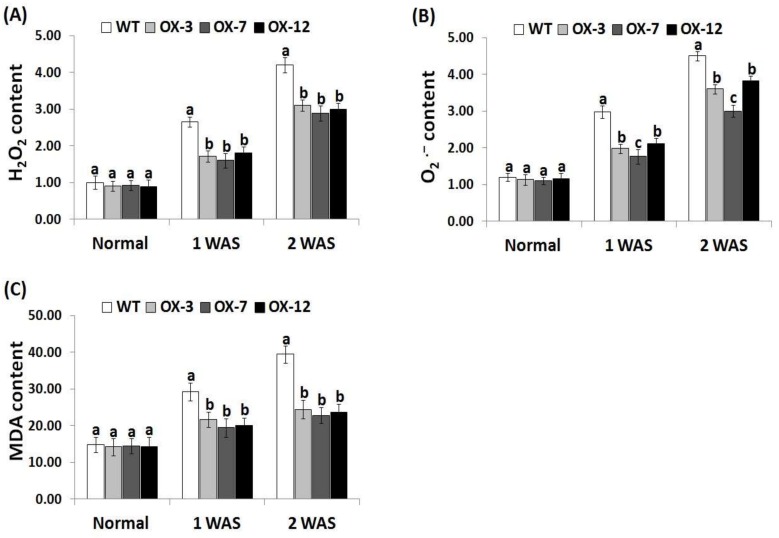
Hydrogen peroxide (H_2_O_2_, µmol g^−1^ FW) content (**A**), superoxide anion (O_2_^.−^, mm g^−1^ FW) content (**B**), and lipid peroxidation (MDA, µmol g^−1^ FW) level (**C**) of the wild type and *StDREB2*-overexpressing cotton lines under normal and drought conditions. WAS represents week after drought stress. Data represent means ± SE (*n* = 5). Same letters on columns tops denote non-significant differences (*p* ≤ 0.05).

Reactive oxygen species are toxic and greatly induce oxidative damage in plant cells [66]. In order to investigate whether *StDREB2* overexpression in cotton could scavenge toxic ROS and alleviate the resulting oxidative damages, the contents of ROS (H_2_O_2_, O_2_^−^) and MDA reflecting the degree of drought stress-induced plant damage were calculated in the wild type and *StDREB2*-overexpressing lines under normal and drought conditions (Figure 5A–C). There were no obvious differences in the contents of H_2_O_2_, O_2_^−^, and MDA between the wild type and overexpressed cotton genotypes under normal condition (Figure 5A–C). By contrast, when exposed to drought conditions, increase in the level of H_2_O_2_, O_2_^−^, and MDA was detected for the wild type and *StDREB2*-overexpressing plants as compared with normal condition. The highest increases were recorded at 2 WAS. Nevertheless, under drought conditions, the wild-type cotton plants significantly accumulated more H_2_O_2_, O_2_^−^, and MDA levels than the transgenic lines (Figure 5A–C). These findings indicate that *StDREB2* overexpression in cotton reduced ROS and MDA level as well as the oxidative damages caused by drought stress, thereby enhancing drought tolerance in transgenic cotton lines. Our findings are also in agreement with previous reports that reported that overexpression of stress-tolerance genes could inhibit membrane damage and significantly reduce ROS and MDA accumulation under stress conditions [28,41].

### 3.6. StDREB2 Overexpression in Cotton Enhances Antioxidant Enzyme Activity under Drought Stress

**Figure 6:**
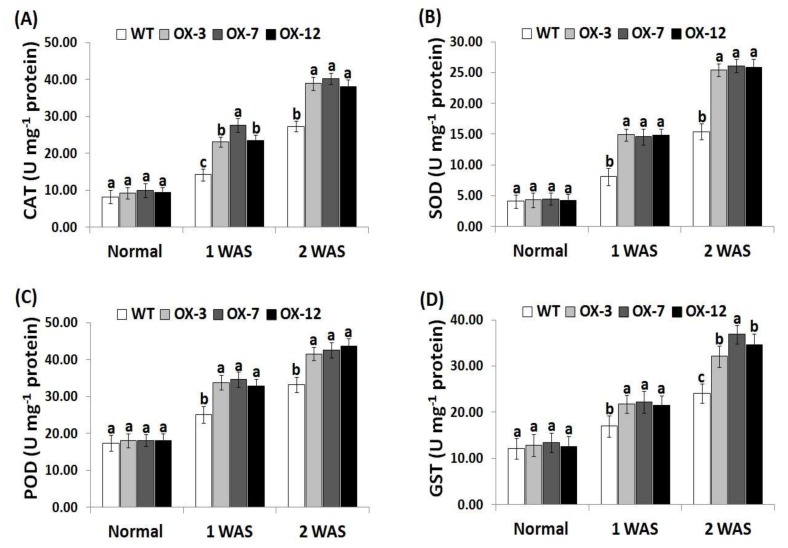
Activities of catalase (CAT) (**A**), superoxide dismutase (SOD) (**B**), peroxidase (POD) (**C**), and glutathione-S-transferase (GST) (**D**) in wild type and *StDREB2*-overexpressing cotton lines under normal and drought conditions. WAS represents week after drought stress. Data represent means ± SE (*n* = 5). Same letters on columns tops denote non-significant differences (*p* ≤ 0.05).

To investigate the causes of the reduced levels of ROS in transgenic cotton plants under drought condition, the activity of four antioxidant enzymes encoding ROS scavenging (CAT, SOD, POD, and GST) was assayed in the wild type and overexpressed genotypes (Figure 6A–D). No obvious difference in these four enzymes’ activities was observed between the wild type and *StDREB2*-overexpressing cotton under normal condition. Upon subjection to drought stress, remarkable increases were recorded in all lines as compared with the normal condition. The highest levels were recorded at 2 WAS. Moreover, these increases were significantly higher in the transgenic lines than in the wild type (Figure 6A–D). The data indicate that *StDREB2* overexpression conferred higher antioxidant enzyme activities to the transgenic cotton lines to counteract the harmful ROS impacts and alleviate the resulting oxidative damages. These findings are also in agreement with previous studies that revealed higher antioxidant enzyme activities in cotton plants overexpressing stress-tolerant genes as compared to the wild-type [38,41].

### 3.7. StDREB2 Overexpression in Cotton Induces Antioxidants and Stress-Related Genes Expression under Drought Stress

**Figure 7:**
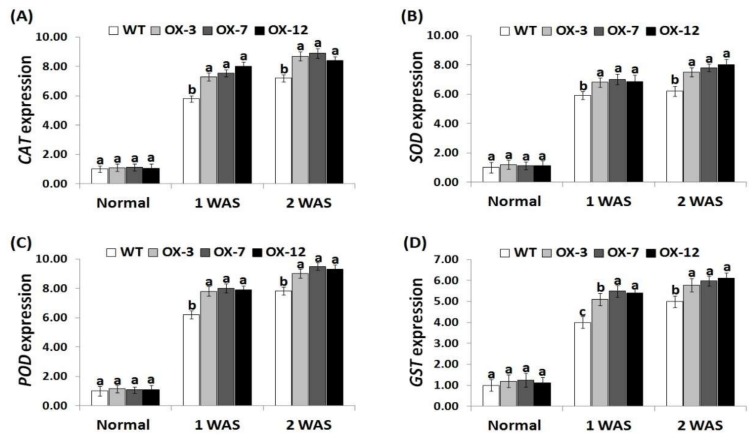
Expression levels of *CAT* (**A**), *SOD* (**B**)*, POD* (**C**), and *GST* (**D**) genes in the wild type and *StDREB2*-overexpressing cotton lines under normal and drought conditions. Data represent means ± SE (*n* = 5). Same letters on columns tops denote non-significant differences (*p* ≤ 0.05).

**Figure 8:**
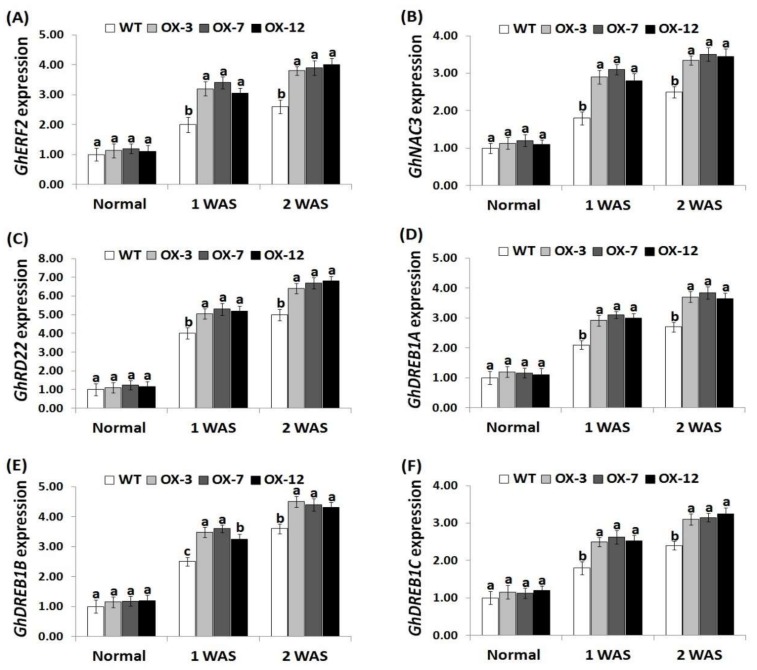
Expression levels of *GhERF2* (**A**), *GhNAC3* (**B**), *GhRD22* (**C**), *GhDREB1A* (**D**), *GhDREB1B* (**E**), and *GhDREB1C* (**F**) genes in the wild type and *StDREB2*-overexpressing cotton lines under normal and drought conditions. Data represent means ± SE (*n* = 5). Same letters on columns tops denote non-significant differences (*p* ≤ 0.05).

To reveal the molecular mechanism of enhanced drought tolerance in *StDREB2*-overexpressing transgenic cotton lines, the expression of four antioxidant genes (*CAT, SOD, POD*, *GST*) and six stress-tolerant genes (*GhERF2, GhNAC3, GhRD22, GhDREB1A, GhDREB1B, GhDREB1C*) were quantified in the wild type and the *StDREB2*-overexpressing cotton grown under normal and drought conditions. No significant difference in the expression levels of antioxidant enzyme genes and stress-responsive genes was observed between lines under normal conditions (Figure 7A–D and Figure 8A–F). Upon exposure to drought conditions, the expression of the analyzed genes were induced as compared to normal condition. *StDREB2*-overexpressing cotton lines revealed higher transcription levels of all genes than the wild type ( 7A–D and 8A–F ). The highest expression levels were recorded at 2 WAS. The results suggest that *StDREB2* overexpression might enhance drought tolerance through up-regulating the expression of genes mediating defense mechanisms and ROS scavenging pathways. Moreover, our results of genes expression are in agreement with that of the antioxidant enzymes activities. These results are also in harmony with the previously published data that revealed higher expression levels of the antioxidant genes (*CAT, SOD, POD*, *GST*) and stress-related genes (*GhERF2, GhNAC3, GhRD22, GhDREB1A, GhDREB1B, GhDREB1C*) in *AmDUF1517*-overexpressing transgenic cotton lines as compared to the wild type under drought condition [41]. Previous studies also documented an additional functional role for *StDREB2* in improving salt and cadmium stress tolerance in potato [35,36].

All the aforementioned results demonstrated the negative effect of drought on the photosynthesis, gas-exchange, chlorophyll, and other physiological mechanisms in cotton plants. These results were also supported by the previous reports that addressed the adverse impacts of water stress on various plant species. These reports exhibited the sensitivity of photosynthesis, chlorophyll content, water use efficiency, and various physiological processes to water stress [67,68,69,70,71]. On the other hand, *StDREB2* overexpression revealed high potential in enhancing drought stress tolerance in cotton crop by modulating such physiological and biochemical attributes.

## 4. Conclusions

Cotton cultivation is affected by drought in dry regions. To augment the drought tolerance of cotton, we overexpressed the potato *StDREB2* gene in cotton, and validated its transcription level in T_0_ and T_3_ transgenic cotton lines using qRT-PCR. Three T_3_ transgenic cotton lines revealing the highest *StDREB2* transcription level were used in drought tolerance analyses. The results showed that *StDREB2* overexpression could augment drought stress tolerance of cotton via the induction of plant biomass, photosynthetic capacity, gas-exchange characteristics, ROS scavenging, antioxidant enzymes activities, osmolytes accumulation, osmotic regulation, and expression of stress-related genes expression. *StDREB2* could be an important candidate gene for enhancing cotton tolerance to drought stress. Further future work should focus on exploring the signaling networks and function of *StDREB2* gene to understand its molecular mechanisms mediating drought stress tolerance in greater depth.
